# The impact of lettuce-derived polyphenols on laying hens: Production performance, antioxidant status, immune function, and cecal microbiota profiles

**DOI:** 10.1016/j.psj.2026.106932

**Published:** 2026-04-13

**Authors:** Xueyuan Jiang, Hulong Lei, Peng Jia, Dong Xia, Haiying Zhao, Zhou Tong, Shiwei Wei, Naisheng Lu

**Affiliations:** a Key Laboratory of Livestock and Poultry Resources (Pig) Evaluation and Utilization of Ministry of Agriculture and Rural Affairs, Shanghai Engineering Research Center of Breeding Pig, Institute of Animal Husbandry and Veterinary Science, Shanghai Academy of Agricultural Sciences, Shanghai 201106, PR China; b Zhejiang Jiameishan Plant Technology Co., Shanghai 201601, PR China; c Shanghai Agrobiological Gene Center, Shanghai 201106, PR China

**Keywords:** Polyphenol-rich lettuce extract (PRLE), Laying hens, Antioxidant, Immunoglobulin, Cecal microbiota

## Abstract

This study aimed to evaluate the effects of dietary supplementation with polyphenol-rich lettuce extract (PRLE) derived from the high-polyphenol lettuce cultivar Binfen-1 (BF-1) on production performance, antioxidant capacity, immune function, and cecal microbiota composition of Hyline Brown laying hens. A total of 140 healthy pre-laying hens (15 days before egg production) were randomly assigned to 4 groups: a control group fed a corn-soybean basal diet, and three experimental groups (TR1, TR2, TR3) fed the basal diet supplemented with 0.05%, 0.1%, and 0.15% PRLE, respectively. Diets were fed for 12 wk, afterwards 10 bird per group were euthanized for sampling blood and cecal digesta. Production performance monitoring showed that compared with the control group, 0.05% and 0.15% PRLE supplementation significantly increased the laying rate during Weeks 1–4 (*p* < 0.05), but no significant effects on laying rate were observed in Weeks 5–8 or 9–12 (*p* > 0.05). For serum antioxidant indices, PRLE supplementation significantly reduced malondialdehyde (MDA) content and increased glutathione peroxidase (GSH-Px) activity across all experimental groups (*p* < 0.05). In terms of immunity, 0.1% PRLE significantly elevated serum immunoglobulin (Ig) G and IgM concentrations (p < 0.05). 16S rRNA gene sequencing revealed that PRLE supplementation increased the relative abundances of *Firmicutes, Desulfobacterota*, and *Actinobacteriota* while decreasing *Proteobacteria* at the phylum level(*p* < 0.05). At the genus level, it reduced the abundance of the conditional pathogen *Peptococcus* and increased the abundances of beneficial bacteria (e.g., *Bacteroides, Lachnospiraceae, Butyricicoccus, Romboutsia*) (*p* < 0.05). PICRUSt2 functional prediction indicated that PRLE enriched pathways related to infectious bacterial diseases, cell motility, and the immune system, while reducing pathways associated with drug resistance and glycan metabolism. Non-targeted metabolomics showed that PRLE significantly altered the cecal metabolite profile and modulated key pathways including arachidonic acid metabolism, bile secretion, and steroid hormone biosynthesis. In conclusion, dietary PRLE supplementation enhances the antioxidant capacity and immune function of Hyline Brown laying hens, improves cecal microbiota composition and metabolic profiles, but exerts only transient (Weeks 1–4) positive effects on laying rate under the tested conditions. This study provides a scientific basis for the application of PRLE in commercial laying hen production, with future research needed to optimize its dosage, supplementation duration, and age-specific efficacy.

## Introduction

The global demand for high-quality protein is increasingly met by the poultry industry, which serves as a primary source of meat and eggs. Driven by advancements in genetic selection, nutritional science, and husbandry management, the poultry sector has achieved remarkable improvements in production efficiency. Nevertheless, persistent challenges-including environmental stressors (e.g., temperature fluctuations, poor air quality) and pathogen infections-continue to hinder the sustainable development of the industry, highlighting the urgent need for innovative strategies to enhance poultry health, production performance, and overall sustainability (Grzinic et al., 2023). In recent years, the incorporation of natural bioactive compounds into poultry diets, particularly for laying hens, has emerged as a promising area of research in poultry nutrition. Polyphenols, a large class of bioactive phytochemicals abundant in plant-based foods (e.g., strawberries, mangoes, apples, rhubarb, tea, onions, and red wine), have attracted significant attention due to their diverse biological activities (Durazzo et al., 2019). To date, over 8,000 distinct phenolic compounds have been identified in plants, with documented physiological functions including antioxidant, anti-inflammatory, antibacterial, immunomodulatory, and gut health-promoting properties (Ji et al., 2019; Bucciantini et al., 2021; Bae et al., 2022; Jiang et al., 2023). These compounds have been shown to exert positive effects on multiple physiological processes in poultry, such as improving gut barrier integrity, enhancing growth performance, reducing lipid peroxidation, and mitigating oxidative stress.

Lettuce (Lactuca sativa L.) is a globally cultivated and consumed vegetable, valued not only for its palatable texture and flavor but also for its nutritional attributes. A growing body of literature has reported the anti-inflammatory potential of polyphenols isolated from lettuce. For instance, polyphenolic extracts from Lactuca sativa var. Maravilla de Verano have been shown to inhibit the lipopolysaccharide (LPS)-induced release of nitric oxide (NO), tumor necrosis factor-α (TNF-α), and interleukin-6 (IL-6) in J774A.1 macrophages (Adesso et al., 2016). Additionally, fermented lettuce polyphenol extracts have been found to alleviate symptoms and reduce serum levels of proinflammatory cytokines (IL-6, IL-1, and TNF-α) in mice with rheumatoid arthritis (Park et al., 2023). The novel lettuce cultivar Binfen-1 (BF-1), developed by the Shanghai Academy of Agricultural Sciences through selective breeding of the common cultivar Lollo Rossa, exhibits a notably high polyphenol content of 7.2 g kg^−1^-substantially higher than that of other reported lettuce cultivars (Cheng et al., 2014; Jiangseubchatveera et al., 2023). Its successful large-scale cultivation across multiple regions further underscores its potential for use in studies investigating nutritional value and biological efficacy. Lu et al. (Lu et al., 2025) employed an integrated approach combining network pharmacology and high-performance liquid chromatography (HPLC) quantification to identify isoquercitrin and chlorogenic acid as the core bioactive components of polyphenol-rich lettuce extract (PRLE). In vitro experiments further demonstrated that PRLE exhibits potent antioxidant and anti-inflammatory activities.

Despite the extensive research on the applications of polyphenols in livestock nutrition, there remains a critical gap in knowledge regarding the effects of PRLE supplementation in laying hen diets. To address this research deficit, the present study was designed to evaluate the impact of dietary PRLE supplementation on the antioxidant capacity, immune function, and cecal microbiota composition of laying hens. The findings of this study aim to provide a scientific basis for the practical application of PRLE in commercial laying hen production.

## Materials and methods

The experimental protocol was reviewed and approved by the Institutional Animal Care and Use Committee (IACUC) of the Shanghai Academy of Agricultural Sciences, with the Ethical Clearance Certificate Number SAASPZ0525139.

### Experimental materials

The lettuce cultivar BF-1 was developed by the Shanghai Academy of Agricultural Sciences via selective breeding of Lollo Rossa. For this study, BF-1 lettuce was cultivated in Jinshan District, Shanghai, from February 2023 to May 2023, under the meticulous care of technical staff from Jiameishan Plant Technology Co., Ltd. PRLE was prepared following the extraction method described by Lu et al. (Lu et al., 2025).

### Experimental animals and husbandry management

A total of 140 healthy Hyline Brown pre-laying hens (approximately 15 days before egg production, purchased from Shanghai Yingfang Egg Chicken Farm), were randomly assigned to 4 treatment groups based on body weight, with 35 hens per group. The control group received a corn-soybean meal basal diet formulated in accordance with the Chinese Layers Feeding Standards (GB/T 5916-2020). The three experimental groups (TR1, TR2, and TR3) were fed the same basal diet supplemented with 0.05%, 0.1%, and 0.15% PRLE, respectively. The PRLE used in this study was provided by Femisci Biotechnology Co., Ltd. (Shanghai, China). All laying hens were housed in fully enclosed three-dimensional wire cages under controlled artificial lighting conditions (7 W LED lamps, 16 hours of light per day) and maintained with optimal ventilation throughout the experimental period. A 1-week adaptation period was implemented before the formal 12-week trial commenced.

### Production performance monitoring

Daily records were kept for the number of eggs produced and individual egg weight. The average laying rate and average egg weight for each group were calculated at 4-week intervals. The laying rate was computed using the formula: Laying rate (%) = (Number of eggs produced / Number of hens) × 100%.

### Egg quality

On the final day of the trial, 20 eggs were selected from each treatment group for egg quality measurements. Eggshell strength, protein height, harshtick and yolk colo were determined using a DET6000 digital egg tester (Nabel Co., Ltd., Japan).

### Sample collection and preparation

At the conclusion of the 12-week experimental period, 10 hens were randomly selected from each group and humanely euthanized via cervical dislocation. Blood samples were collected from the jugular vein into 10 mL centrifuge tubes, allowed to stand at room temperature for 2–3 hours, and then centrifuged at 3000 rpm for 15 minutes to separate serum. The separated serum was stored at −80°C until further analysis. Following blood collection, the abdominal cavity of each hen was dissected, and fresh cecal digesta were collected, immediately frozen in liquid nitrogen, and stored at −80°C for subsequent microbiota and metabolomic analyses.

### Determination of antioxidant indices and immunoglobulin levels

Commercial assay kits (Nanjing Jiancheng Bioengineering Institute, Nanjing, China) were used to measure the serum activities of superoxide dismutase (SOD) and glutathione peroxidase (GSH-Px), as well as the serum contents of total antioxidant capacity (T-AOC) and malondialdehyde (MDA). Serum concentrations of immunoglobulin G (IgG), immunoglobulin A (IgA), and immunoglobulin M (IgM) were determined using an enzyme-linked immunosorbent assay (ELISA) with commercial kits (Cusabio Biotech Co., Ltd., Wuhan, China), and absorbance values were measured using a microplate reader.

### Cecal microbiota analysis

Total genomic DNA was extracted from cecal digesta samples using a commercial stool DNA extraction kit (Ultra Clean Fecal DNA Isolation Kit, Solarbio Co., Ltd., Beijing, China). The V4-V5 hypervariable regions of the bacterial 16S rRNA gene were amplified using the specific primers 515F (5′-GTGCCAGCMGCCGCGG-3′) and 907R (5′-CCGTCAATTCMTTTRAGTTT-3′). Library construction and high-throughput sequencing were performed by Majorbio Bio-Pharm Technology Co., Ltd. (Shanghai, China) using the Illumina MiSeq PE250 platform. All obtained raw sequence datasets have been uploaded to the NCBI Sequence Read Archive (SRA) with the accession number PRJNA1344448. Raw sequencing data were processed and analyzed using the Majorbio Cloud platform (www.majorbio.com).

### Non-targeted metabolomics analysis of cecal metabolites

For the LC-MS analysis, a Thermo UHPLC-Q Exactive HF-X system was employed in conjunction with an ACQUITY HSS T3 column (Waters Corporation, Milford, Connecticut, USA). Two mobile phase compositions were utilized: the first consisted of 0.1% formic acid in water and acetonitrile at a 95:5 volume ratio, while the second comprised 0.1% formic acid in acetonitrile, isopropanol, and water at a 47.5:47.5:5 volume ratio. The column temperature was set at 40 °C, the flow rate was set at 0.40 mL/min, and the mass spectral signals of the samples were collected in positive and negative ion scanning modes. The entire LC-MS experimental workflow was executed by Shanghai Majorbio Co., Ltd. (Shanghai, China; accessible at https://www.majorbio.com/) following standardized operational protocols.

### Statistical analysis

All experimental data were analyzed using one-way analysis of variance (ANOVA) with SPSS 20.0 software. Multiple comparisons between groups were conducted using Duncan’s multiple range test. Results are presented as “Mean ± Standard Error of the Mean (SEM)”. A probability value of *p* < 0.05 was considered statistically significant, while *p* > 0.05 indicated no significant difference. Histograms were generated using GraphPad Prism 8.0 software.

## Results

### Effects of PRLE on laying rate and average egg weight

As shown in Table 1, compared with the control group, dietary supplementation with 0.05% (TR1) and 0.15% (TR3) PRLE significantly increased the laying rate of hens during the first 4 weeks of the trial (*p* < 0.05). However, no significant differences in laying rate were observed among the four groups in the subsequent experimental periods (Weeks 5–8 and Weeks 9–12; *p* > 0.05). Throughout the entire 12-week trial, there were no significant differences in average egg weight between the control group and the PRLE-supplemented groups (*p* > 0.05).

**Table 1:** Effects of dietary PRLE supplementation on laying rate and average egg weight in laying hens.

	Control	TR1	TR2	TR3	SEM	*p* Value
Laying rate (%)						
Wk 1-4	25.64^b^	30.77^a^	26.67^b^	32.05^a^	0.83	0.032
Wk 5-8	75.64	76.92	81.11	71.79	3.23	0.141
Wk 9-12	80.77	82.05	81.11	79.81	2.41	0.203
Average egg weight (g)						
Wk 1-4	51.85	52.40	52.31	52.83	2.75	0.724
Wk 5-8	58.36	57.32	58.82	57.88	8.30	0.587
Wk 9-12	60.11	59.76	59.74	60.25	1.37	0.961

a,b Means in the same row with different letters are significantly different (*p* < 0.05). n = 20. The same below.

### Effects of PRLE on egg quality

As shown in Table 2, there were no significant differences in eggshell strength, protein height, Haugh units and yolk color among the different groups (*p* > 0.05). The results indicate that after a feeding period of 3 months, dietary PRLE supplementation did not have any impact on egg quality.

**Table 2:** Effects of dietary PRLE supplementation on egg quality in laying hens.

	Control	TR1	TR2	TR3	SEM	*p* Value
Eggshell strength (N)	5.19	5.41	5.54	5.50	0.81	0.327
Protein height (mm)	9.07	9.21	9.43	9.48	0.64	0.172
Haugh units	93.56	95.27	96.43	96.61	4.21	0.282
yolk color	6.3105	6.2316	6.1429	6.0333	0.40	0.183

### Effects of PRLE on serum antioxidant indices

**Fig. 1:**
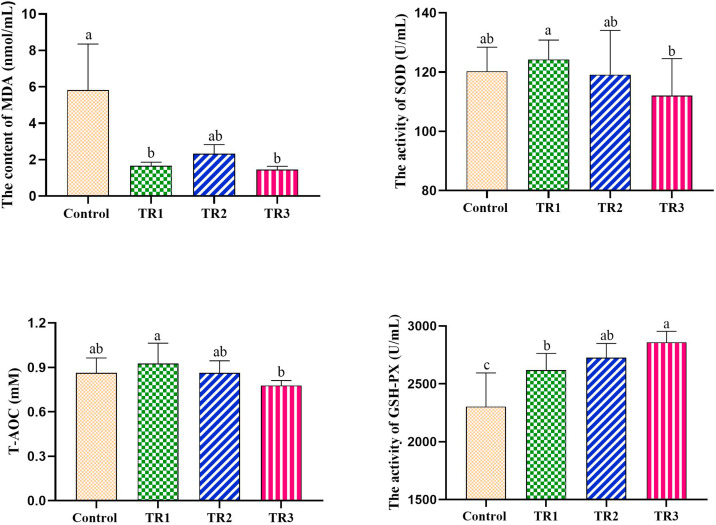
Effects of dietary PRLE supplementation on antioxidant indices in serum of laying hens. Abbreviations: MDA, malondialdehyde; SOD, superoxide dismutase; T-AOC, total antioxidant capacity; GSH-Px, glutathione peroxidase. Data are expressed as the means ± SEMs, n = 10. a,b,c mean values with different superscript letters within groups were significantly different (*p* < 0.05).

**Fig. 2:**
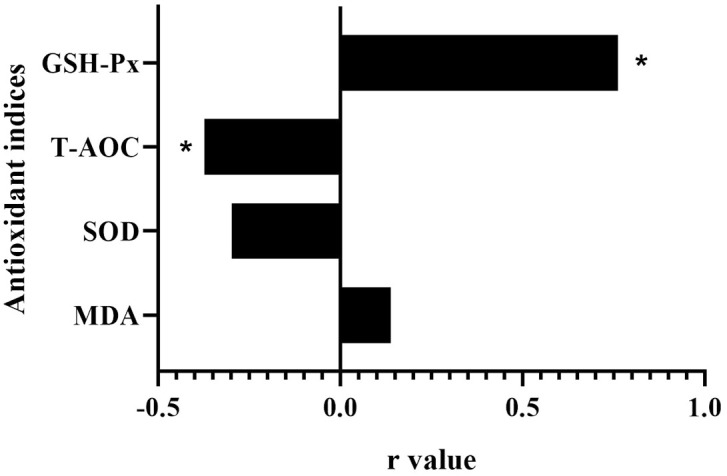
Spearman correlation analysis between the antioxidant indices and the level of PRLE supplementation. n = 10, * means p < 0.05.

As illustrated in Fig. 1, compared with the control group, dietary PRLE supplementation significantly reduced serum malondialdehyde (MDA) content (p < 0.05) and increased serum glutathione peroxidase (GSH-Px) activity (*p* < 0.05) across all supplemented groups. Notably, when comparing the 0.05% PRLE group (TR1) with the 0.15% PRLE group (TR3), the latter exhibited significantly lower serum superoxide dismutase (SOD) activity and total antioxidant capacity (T-AOC) (*p* < 0.05), alongside a further increase in GSH-Px activity (*p* < 0.05). We conducted a correlation analysis between the antioxidant indices and the level of PRLE supplementation, as shown in Fig. 2. Spearman correlation analysis revealed that the PRLE supplementation level was significantly positively correlated with GSH-Px activity (r = 0.762, *p* < 0.01) and significantly negatively correlated with T-AOC (r = −0.373, *p* < 0.05).

### Effects of PRLE on serum immunoglobulin concentrations

**Fig. 3:**
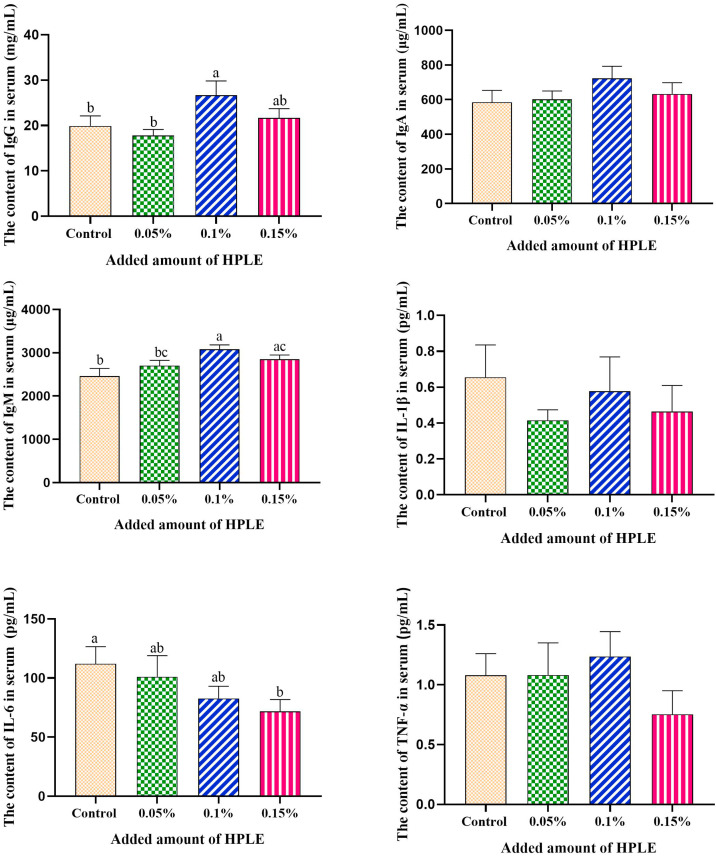
Effects of dietary PRLE supplementation on immunoglobulin concentration in serum of laying hens. Data are expressed as the means ± SEMs, n = 10. a,b,c mean values with different superscript letters within groups were significantly different (*p* < 0.05).

Fig. 3 presents the serum concentrations of immunoglobulin A (IgA), immunoglobulin G (IgG), and immunoglobulin M (IgM) in laying hens fed the control diet or diets supplemented with different concentrations of PRLE. Compared with the control group, supplementation with 0.1% PRLE (TR2) significantly increased serum IgG and IgM concentrations, and 0.15% PRLE (TR3) significantly increased serum IgM concentrations (*p* < 0.05). In contrast, there were no significant differences in serum IgA concentrations among the four treatment groups (*p* > 0.05).

### Effects of PRLE on cecal microbiota diversity

**Fig. 4:**
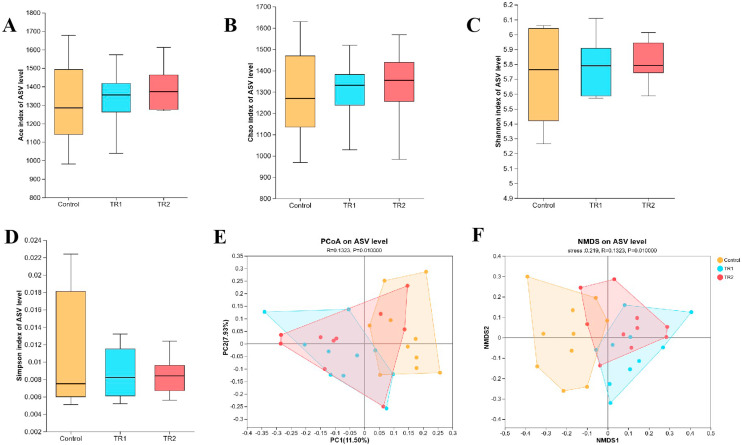
Effects of dietary PRLE supplementation on cecal microbiota diversity in laying hens. Bacterial richness was estimated by the Ace (A) and Chao (B); Bacterial diversity was assessed by the Shannon index (C) and Simpson index (D); (E) Two-dimensional OTU abundance-based principal coordinate analysis (PCoA) of cecal microbiota; (F) Non-metric multidimensional scaling (NMDS) of cecal microbiota.

A total of 1,623,632 high-quality sequences were obtained from all cecal digesta samples, with an average sequence length of 418 bp. Based on a 97% nucleotide sequence identity threshold, 1602 operational taxonomic units (OTUs) were identified. As shown in Figures 4A–D, dietary PRLE supplementation did not significantly affect the alpha diversity of the cecal microbiota (*p* > 0.05), as indicated by the Ace index (richness), Chao index (richness), Shannon index (diversity), and Simpson index (diversity). Principal Coordinate Analysis (PCoA, Fig. 4E) and Non-Metric Multidimensional Scaling (NMDS, Fig. 4F) were employed to assess beta diversity (similarities and differences in microbiota composition between groups). The results revealed a clear separation in cecal microbiota composition between the control group and the PRLE-supplemented groups (*p* < 0.05), while no significant separation was observed between the different PRLE-supplemented groups (*p* > 0.05).

### Effects of PRLE on Cecal Microbiota Composition

**Fig. 5:**
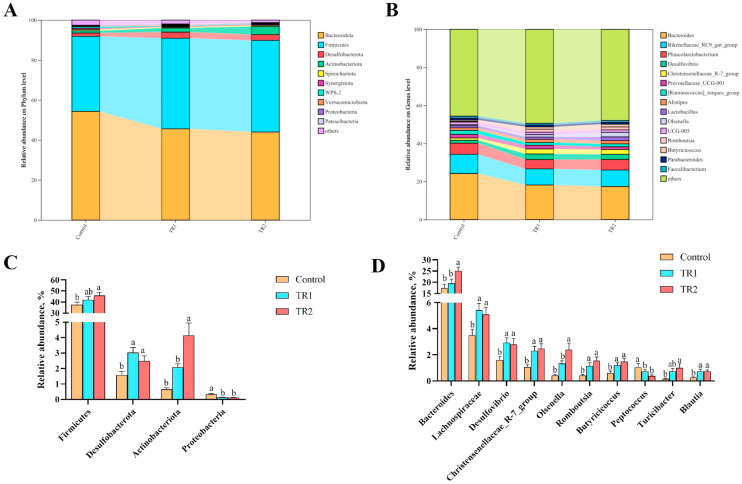
Effect of dietary PRLE supplementation on cecal microbiota composition of laying hens. (A) Microbial composition at the phylum level; (B) Microbial composition at the genus level; (C) Differences among treatments at the phylum levels; (D) Differences among treatments at the genus levels.

The relative abundances of cecal bacteria at the phylum and genus levels are presented in Fig. 5. At the phylum level (Fig. 5A), *Bacteroidota* and *Firmicutes* were the dominant phyla in the cecal microbiota of all groups. As shown in Fig. 5C, compared with the control group, PRLE supplementation significantly increased the relative abundances of *Firmicutes, Desulfobacterot*a, and *Actinobacteriota* while decreasing the relative abundance of *Proteobacteria* (*p* < 0.05). At the genus level (Fig. 5B), *Bacteroides, Rikenellaceae_RC9_gut_group, Phascolarctobacterium, Desulfovibrio, Christensenellaceae_R-7_group,* and *Prevotellaceae_UCG-001* were the most abundant genera in cecal digesta. Compared with the control group, PRLE-supplemented groups exhibited significantly lower relative abundances of *Peptococcus* and significantly higher relative abundances of *Bacteroides, Lachnospiraceae, Desulfovibrio, Christensenellaceae_R-7_group, Olsenella, Romboutsia, Butyricicoccus, Turicibacter,* and *Blautia* (*p* < 0.05; Fig. 4D).

**Fig. 6:**
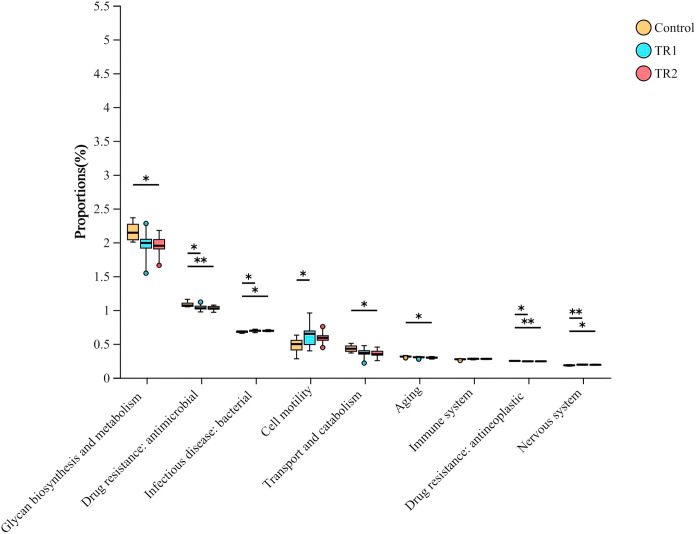
Prediction of microbial function in the cecum of laying hens. The second level of the KEGG pathway is shown in the extended error bar.

The functional potential of the cecal bacterial community was predicted using PICRUSt2 analysis based on 16S rRNA gene sequencing data. At the second level of the KEGG pathway hierarchy, nine differentially enriched functional pathways were identified among the treatment groups (Fig. 6). Compared with the control group, the PRLE-supplemented groups exhibited significant enrichment in pathways related to infectious bacterial diseases, cell motility, the immune system, and the nervous system (*p* < 0.05). Additionally, PRLE supplementation significantly reduced the enrichment of pathways associated with antimicrobial drug resistance, antineoplastic drug resistance, glycan biosynthesis and metabolism, and transport and catabolism (*p* < 0.05).

### Effects of PRLE on cecal metabolites

**Fig. 7:**
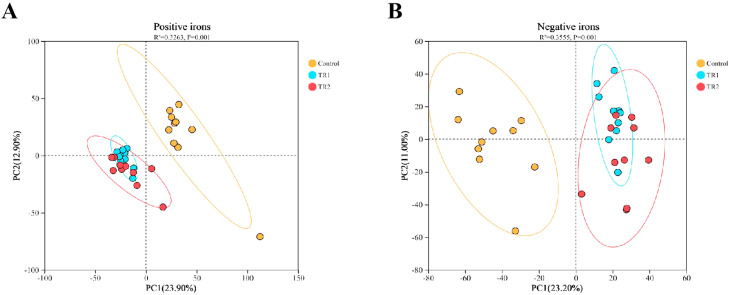
Principal component analysis (PCA) scores of cecum metabolites. Includes positive (A) and negative (B) ion modes. Model parameters for control, TR1 and TR2 groups (positive ions, R^2^ = 0.3263, *p* = 0.001; negative ions, R^2^ = 0.3555, *p* = 0.001).

To explore whether PRLE-induced changes in cecal microbiota were associated with altered intestinal metabolite profiles, a non-targeted metabolomics approach based on LC-MS/MS was used to analyze the metabolite composition of cecal digesta. The PCA score plots (Fig. 7) clearly showed distinct separation of cecal metabolites between the control group and the PRLE-supplemented groups under both positive and negative ion modes. The model parameters for the three groups were as follows: positive ion mode, R^2^ = 0.3263, *p* = 0.001; negative ion mode, R^2^ = 0.3555, *p* = 0.001. These results confirm that dietary PRLE supplementation significantly altered the cecal metabolite profile of laying hens.

**Fig. 8:**
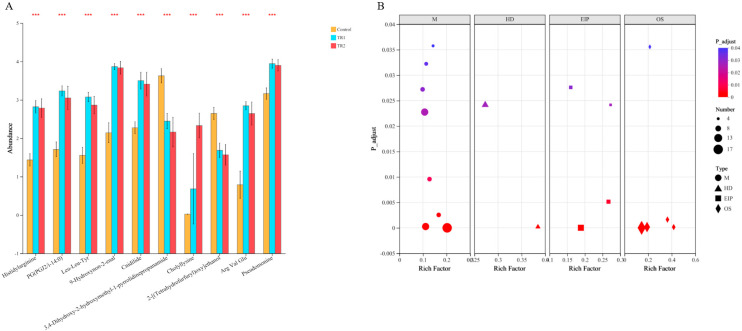
Effect of dietary PRLE supplementation on metabolites in the cecum of laying hens. (A) differential metabolites; (B) KEGG pathway enrichment analysis. *** (p < 0.001).

Differentially abundant metabolites and their associated metabolic pathways are presented in Fig. 8. Compared with the control group, dietary PRLE supplementation significantly increased the cecal contents of histidylarginine, PG(PGJ2/i-14:0), leu-Leu-Tyr, 9-Hydroxynon-2-enal, cnidilide, cholyllysine, Arg Val Glu, and pseudomonine, while significantly decreasing the contents of 3,4-Dihydroxy-2-hydroxymethyl-1-pyrrolidinepropanamide and 2-[(Tetrahydrofurfuryl)oxy]ethanol (*p* < 0.05). Further pathway enrichment analysis revealed that PRLE supplementation significantly altered metabolic pathways including arachidonic acid metabolism, bile secretion, neuroactive ligand-receptor interaction, and steroid hormone biosynthesis.

### Correlation analysis of Cecal Microbes and Metabolites

**Fig. 9:**
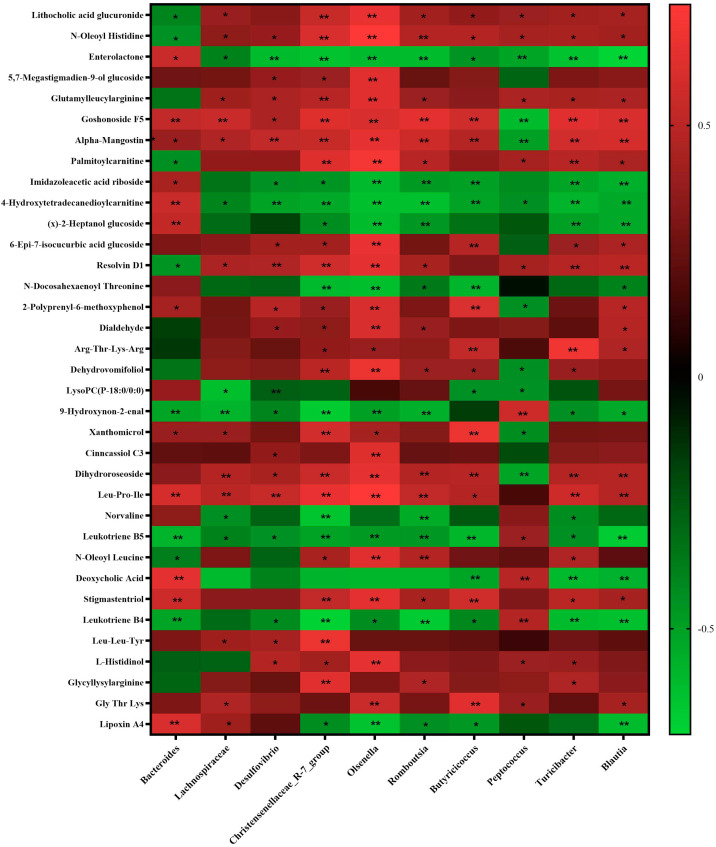
Spearman correlation analysis between microbes and metabolite profiles*.* Only significant enriched microbes and significant differential metabolites were analysised. n = 10. * means *p* < 0.05, ** indicates *p* < 0.01.

To explore the intrinsic links between microbial shifts and metabolite profiles, we conducted a Spearman correlation analysis (|r| > 0.6, *p* < 0.01), and the data presented in Fig. 9. Several beneficial bacterial genera enriched in the PRLE group showed significant positive correlations with a series of bioactive metabolites. For example, *Olsenella* exhibited strong positive correlations with N—Oleoyl Histidine (r = 0.74, *p* < 0.01), Goshonoside F5 (r = 0.60, *p* < 0.01), Alpha-Mangostin (r = 0.65, *p* < 0.01), Dehydrovomifoliol (r = 0.67, *p* < 0.01), and Dihydroroseoside (r = 0.65, *p* < 0.01). *Lachnospiraceae* showed significant positive correlations with Goshonoside F5 (r = 0.55, *p* < 0.01), Resolvin D1 (r = 0.45, *p* < 0.05), and Dihydroroseoside (r = 0.47, *p* < 0.01). *Bacteroides* was significantly positively correlated with Goshonoside F5 (r = 0.52, *p* < 0.01) and 2-Polyprenyl-6-methoxyphenol (r = 0.43, *p* < 0.05). *Butyricicoccus* showed strong positive correlations with metabolites such as N—Oleoyl Histidine (r = 0.47, *p* < 0.01), Goshonoside F5 (r = 0.57, *p* < 0.01), 2-Polyprenyl-6-methoxyphenol (r = 0.61, *p* < 0.01), and Xanthomicrol (r = 0.67, *p* < 0.01). In contrast, the conditional pathogen *Peptococcus* showed significant negative correlations with metabolites possessing antioxidant and anti-inflammatory properties, such as Goshonoside F5 (r = −0.61, *p* < 0.01), Alpha-Mangostin (r = −0.50, *p* < 0.01), Dehydrovomifoliol (r = −0.44, *p* < 0.05), and Dihydroroseoside (r = −0.52, *p* < 0.01). Conversely, it showed significant positive correlations with Deoxycholic Acid (r = 0.49, *p* < 0.01), Leukotriene B5 (r = 0.40, *p* < 0.05), and Leukotriene B4 (r = 0.47, *p* < 0.01).

## Discussion

Polyphenols are widely recognized for their diverse biological activities, including antioxidant, anti-inflammatory, antibacterial, anticancer, and antistress properties (Cueva et al., 2020). The core active substances in PRLE are isoquercitrin and chlorogenic acid (Lu et al., 2025), which have been previously shown to improve animal growth performance, immune function, and intestinal barrier integrity by enhancing anti-inflammatory, antibacterial, and antioxidant capacities, as well as regulating lipid metabolism (Chen et al., 2018, 2018). The present study systematically evaluated the effects of dietary PRLE supplementation on the antioxidant capacity, immune function, and cecal microbiota of Hyline Brown laying hens, with a focus on clarifying its potential as a natural feed additive.

### PRLE and laying hen production performance

Previous studies have reported that dietary polyphenol supplementation can improve the production performance of laying hens. For example, research has found that supplementation with 0.04% quercetin increased the production performance of laying hens (Liu et al., 2023). Similarly, adding 400 mg/kg quercetin to the diet of 52-week-old Tianfu laying hens tended to increase laying rate, feed intake, and egg weight, while reducing the feed-to-egg ratio (Amevor et al., 2021). However, in the present study, PRLE supplementation only significantly increased the laying rate during the initial 4 weeks (Weeks 1–4) of the trial, with no significant effects on laying rate in the middle (Weeks 5–8) or late (Weeks 9–12) stages, nor on average egg weight and egg quality throughout the entire 12-week period. In addition, egg quality parameters, including eggshell strength, protein height, harshtick and yolk color, were not significantly affected by PRLE supplementation, indicating that the overall internal quality of the eggs remained largely intact. We hypothesize that the duration of PRLE administration used in this experiment may not have been sufficient to elicit significant benefits under the specific conditions tested. This discrepancy may be attributed to the fact that the age of the laying hens used in this study (17 weeks at the start of the trial) places them in the early laying stage, during which their physiological status (e.g., reproductive system development, hormone balance) differs significantly from that of hens in the late laying stage (≥50 weeks). Most studies reporting positive effects of polyphenols on egg production and quality have focused on hens in the late laying stage (Kara et al., 2015; Yuan et al., 2016), whereas studies involving early-stage hens often show non-significant or even negative effects (Zhu et al., 2020). This suggests that the response of laying hens to dietary polyphenols may be age-dependent. Additionally, the 12-week trial duration may have been insufficient to observe long-term effects, as some benefits of polyphenols may require a longer period to manifest.

### PRLE and serum antioxidant capacity

Natural plant-derived antioxidants have gained significant attention in poultry nutrition due to their ability to alleviate oxidative stress. Previous studies have demonstrated that quercetin and chlorogenic acid-core components of PRLE-exert potent antioxidant effects. For example, research has reported that supplementation with hesperidin, naringin, or quercetin increased serum SOD and GSH-Px activities while decreasing MDA content in 28-week-old Lohmann White laying hens (Iskender et al., 2016). Similarly, Liu et al. (2023) found that quercetin supplementation enhanced serum SOD and GSH-Px activities and reduced MDA levels in laying hens, and Arslan et al. (2022) observed that quercetin decreased hepatic MDA content and alleviated oxidative stress in quails. Chlorogenic acid has also been shown to improve the antioxidant capacity of broilers by increasing the activities of antioxidant enzymes such as SOD, catalase (CAT), and GSH-Px (Liu et al., 2022; Wang et al., 2022; Pan et al., 2023; Zha et al., 2023).

Consistent with these previous findings, the present study showed that PRLE supplementation significantly increased serum GSH-Px activity and reduced serum MDA content in laying hens. GSH-Px is a key antioxidant enzyme that scavenges hydrogen peroxide and lipid peroxides, while MDA is a stable end product of lipid peroxidation and a reliable indicator of oxidative stress levels. The increase in GSH-Px activity and decrease in MDA content confirm that PRLE can enhance the antioxidant capacity of laying hens and mitigate oxidative stress. Notably, the PRLE supplementation level was significantly positively correlated with GSH-Px activity and significantly negatively correlated with T-AOC. This observation warrants further investigation to determine the optimal PRLE concentration for maximizing antioxidant benefits.

### PRLE and serum immunoglobulin levels

Serum immunoglobulin levels are key indicators of an organism’s immune status. Among these, IgG is the most abundant immunoglobulin in humoral immunity, accounting for approximately 75% of total serum immunoglobulins, and plays a critical role in defending against extracellular pathogens. IgM, as the first antibody produced during the initial immune response, is also essential for early infection resistance. In the present study, supplementation with 0.1% PRLE significantly increased serum IgG and IgM concentrations, while having no significant effect on IgA levels. This suggests that PRLE can enhance the humoral immune function of laying hens, particularly by boosting the production of IgG and IgM. The immune-enhancing effect of PRLE may be attributed to its core components, isoquercitrin and chlorogenic acid. Previous studies have shown that quercetin can regulate the proliferation and differentiation of immune cells (e.g., lymphocytes, macrophages) and promote the secretion of cytokines and immunoglobulins (Liu et al., 2023). Chlorogenic acid has also been reported to enhance immune function by activating the NF-κB signaling pathway and increasing the production of proinflammatory cytokines (e.g., TNF-α, IL-6) and immunoglobulins (Chen et al., 2018). By enhancing immune function, PRLE can improve the disease resistance of laying hens and promote overall health.

### PRLE and cecal microbiota

Polyphenols are only partially absorbed in the small intestine; the unabsorbed fraction enters the hindgut, where it is metabolized by gut microbiota. These microbial metabolites (e.g., phenolic acids, flavonoid aglycones) often exhibit higher bioavailability than the parent polyphenols and can further modulate gut microbiota composition (Scalbert and Williamson, 2000). The bidirectional interaction between polyphenols and gut microbiota plays a crucial role in maintaining host health (Gowd et al., 2019), and numerous studies have shown that polyphenol-rich compounds exert antioxidant and anti-inflammatory effects by regulating gut microbiota (Pandey and Rizvi, 2009; Gowd et al., 2019). In rats, polyphenol supplementation has been shown to alter the composition of intestinal microbiota (Larrosa et al., 2009; Viveros et al., 2011). And feeding broilers grape pomace extract or grape seed extract increased the abundance of beneficial ileal bacteria (e.g., *Enterococcus*) and decreased the abundance of potential pathogens (e.g., *Clostridium*) (Viveros et al., 2011). In the present study, PCoA and NMDS analyses revealed a significant separation in cecal microbiota composition between the control group and the PRLE-supplemented groups, indicating that PRLE can alter the overall structure of the cecal microbiota. At the phylum level, PRLE supplementation increased the relative abundances of *Firmicutes, Desulfobacterota*, and *Actinobacteriota* while decreasing the relative abundance of *Proteobacteria. Proteobacteria* is a phylum that includes many pathogenic bacteria (e.g., *Salmonella, Escherichia coli*) and is often associated with intestinal inflammation and dysbiosis (Koren et al., 2012). An abnormal increase in Proteobacteria has been identified as a potential diagnostic marker for gut microbiota imbalance and intestinal epithelial dysfunction (Litvak et al., 2017). The reduction in *Proteobacteria* abundance following PRLE supplementation suggests that PRLE may help maintain intestinal homeostasis and reduce the risk of pathogenic infections.

At the genus level, PRLE supplementation decreased the relative abundance of *Peptococcus*-a conditional pathogen that often causes mixed infections with other bacteria (e.g., *Bacteroides, Streptococcus*)-and increased the abundance of beneficial genera such as *Bacteroides, Lachnospiraceae, Desulfovibrio, Christensenellaceae_R-7_group, Olsenella, Romboutsia, Butyricicoccus, Turicibacter,* and *Blautia. Lachnospiraceae* and *Butyricicoccus* are well-known butyrate-producing genera. Butyrate is the primary energy source for intestinal epithelial cells, plays a critical role in maintaining intestinal barrier integrity, and inhibits intestinal inflammation (Medvecky et al., 2018; Zou et al., 2019; Xie et al., 2021). Studies has found that the combination of chlorogenic acid and epigallocatechin gallate increased the abundance of *Lachnospiraceae*, thereby improving intestinal barrier integrity (Wei et al., 2023). The increase in *Lachnospiraceae* and *Butyricicoccus* abundance in the present study suggests that PRLE may promote butyrate production, which in turn enhances intestinal health. *Bacteroides* is a dominant genus in the mammalian gut and is involved in the digestion and metabolism of dietary polysaccharides, the synthesis of short-chain fatty acids (SCFAs), and the maintenance of intestinal environment stability (Jandhyala et al., 2015; Rowland et al., 2018). *Bacteroides* can also inhibit the growth of pathogenic bacteria by producing antimicrobial peptides (Collins et al., 2016). Studies have found that chlorogenic acid supplementation increased the relative abundance of *Bacteroidaceae* (Wang et al., 2019), which is consistent with the present results. The increase in *Bacteroides* abundance indicates that PRLE may improve nutrient digestion and absorption and enhance intestinal barrier function. *Romboutsia* has been shown to enhance immune function in broilers by activating the intestinal TLR2/NF-κB signaling pathway and improving gut microbiota composition (Song et al., 2024). The increase in *Romboutsia* abundance following PRLE supplementation may contribute to the observed improvement in immune function. While *Desulfovibrio* has been associated with inflammatory bowel disease (Rowan et al., 2010) and metabolic diseases (Petersen et al., 2019), recent studies have suggested that its role is not entirely detrimental. There are studies showing that the relative abundance of *Desulfovibrio* is positively correlated with gut microbiota diversity, which is beneficial for microbiota stability and host health (Le Chatelier et al., 2013). The increase in *Desulfovibrio* abundance in the present study may reflect an improvement in gut microbiota diversity and stability, although further studies are needed to confirm its specific role.

To further explore the functional implications of PRLE-induced changes in cecal microbiota, PICRUSt2 analysis was used to predict microbial functions. The results showed that PRLE supplementation reduced the enrichment of pathways related to antimicrobial and antineoplastic drug resistance, suggesting that PRLE may inhibit the development of drug resistance in intestinal bacteria and reduce the risk of antibiotic resistance gene transfer. Additionally, PRLE supplementation decreased the enrichment of pathways involved in glycan biosynthesis and metabolism, as well as transport and catabolism. These pathways are involved in the metabolism of carbohydrates and the transport of nutrients and metabolites, respectively. The changes in these pathways may be related to altered energy utilization and nutrient absorption in laying hens, although the specific mechanisms require further validation due to the limitations of functional prediction based on 16S rRNA sequencing data.

### PRLE and cecal metabolites

The functional role of gut microbiota is closely linked to their metabolic products. Many microbial metabolites are biologically active and can regulate host cell differentiation, migration, proliferation, and apoptosis, thereby exerting physiological or pathological effects on the host. The non-targeted metabolomics analysis revealed that PRLE supplementation significantly altered the cecal metabolite profile of laying hens, with changes in metabolites such as histidylarginine, PG (PGJ2/i-14:0), and leu-Leu-Tyr. Further pathway analysis showed that PRLE affected key metabolic pathways including arachidonic acid metabolism, bile secretion, neuroactive ligand-receptor interaction, and steroid hormone biosynthesis. These pathways are involved in inflammatory regulation, lipid metabolism, and neuroendocrine function, respectively, suggesting that PRLE may regulate intestinal health and overall physiological function of laying hens by modulating these metabolic pathways. For example, arachidonic acid metabolism is closely associated with inflammatory responses, and changes in arachidonic acid metabolites (e.g., prostaglandins) can affect the severity of intestinal inflammation. Bile secretion plays a critical role in lipid digestion and absorption, and alterations in bile acid metabolism can influence nutrient utilization and gut microbiota composition. Neuroactive ligand-receptor interaction is involved in the regulation of gut-brain communication, which affects intestinal motility and secretion. Steroid hormone biosynthesis is related to reproductive function, which may indirectly affect the production performance of laying hens. The modulation of these pathways by PRLE highlights its potential as a multifunctional feed additive for improving the health and productivity of laying hens.

### PRLE, microbiota functions, and cecal metabolites

To explore the intrinsic relationship between cecal microbial changes and metabolites, we conducted a Spearman correlation analysis. These associations not only confirm the direct impact of the remodeling of the microbiota structure on the metabolic environment, but also suggest a potential mechanism by which PRLE may affect the health of laying hens by regulating specific microbiota. In the present study, several beneficial bacterial genera enriched in the PRLE group formed strong positive correlations with a series of bioactive metabolites, constituting a potential network for beneficial metabolic regulation. For instance, *Olsenella* showed strong positive correlations with N—Oleoyl Histidine, Goshonoside F5, Alpha-Mangostin, and Dihydroroseoside. These metabolites are known for their antioxidant and anti-inflammatory activities (Yu et al., 2019). This association suggests that PRLE may promote the growth of Olsenella, thereby increasing the accumulation of metabolites with anti-inflammatory potential. *Lachnospiraceae* exhibited a significant positive correlation with Resolvin D1. *Lachnospiraceae* is an important butyrate-producing genera. Resolvin D1 is a specialized pro-resolving mediator derived from ω−3 fatty acids and plays a central role in inflammation resolution. Previous studies have confirmed that butyrate can promote macrophage production of Resolvin D1 (Wang et al., 2022). Therefore, this positive correlation indicates that PRLE may enrich butyrate-producing bacteria like *Butyricicoccus* and *Lachnospiraceae*, increase butyrate levels, and subsequently upregulate pro-resolving mediators like Resolvin D1, representing a potential key metabolic pathway for its anti-inflammatory effects. *Peptococcus*, a conditional pathogen, showed strong negative correlations with metabolites possessing antioxidant and anti-inflammatory properties, such as Goshonoside F5, Alpha-Mangostin, Dehydrovomifoliol, and Dihydroroseoside. Conversely, it showed positive correlations with pro-inflammatory mediators Leukotriene B5 and Leukotriene B4. This indicates that the over-proliferation of Peptococcusmay inhibit the transformation or accumulation of certain plant-derived beneficial components. In our research, PRLE may indirectly promote the generation of such beneficial metabolites and inhibit the expression of inflammatory mediators by suppressing *Peptococcus*.

The correlation analysis between microbes and metabolites suggests that PRLE may exert its effects through a dual mechanism: (1) a "gain-of-function" effect, promoting the proliferation of beneficial bacteria and increasing beneficial metabolites such as natural plant active components (Goshonoside F5, Alpha-Mangostin, Dehydrovomifoliol, Dihydroroseoside), anti-inflammatory mediators (Resolvin D1), and bioactive peptides (Leu-Pro-Ile); and (2) a "harm reduction" effect, inhibiting conditional pathogens like *Peptococcusand* facilitating the conversion or reduction of potentially harmful substances by beneficial bacteria.

## Conclusions

In conclusion, the present study demonstrates that dietary supplementation with PRLE enhances the antioxidant capacity and immune function of Hyline Brown laying hens, as evidenced by increased serum GSH-Px activity, reduced serum MDA content, and elevated serum IgG and IgM concentrations. However, PRLE had limited effects on production performance under the tested conditions, with significant improvements in laying rate only observed during the initial 4 weeks of the trial. Additionally, 16S rRNA gene sequencing and non-targeted metabolomics analyses revealed that PRLE alters the composition and function of the cecal microbiota, as well as the cecal metabolite profile, thereby promoting intestinal health of laying hens. This study provides a foundation for understanding the effects of PRLE on commercial laying hens, while emphasizing the need for further research. Future studies should aim to optimize the dosage and duration of PRLE supplementation, explore age-specific responses, and uncover the mechanisms underlying its influence on egg quality and antioxidant capacity. By addressing these research gaps, the practical application of PRLE in poultry nutrition can be further optimized, contributing to the development of sustainable and healthy laying hen production systems.

## CRediT authorship contribution statement

**Xueyuan Jiang:** Writing – review & editing, Writing – original draft, Project administration, Methodology, Formal analysis, Data curation, Conceptualization. **Hulong Lei:** Investigation. **Peng Jia:** Visualization. **Dong Xia:** Supervision, Conceptualization. **Haiying Zhao:** Resources. **Zhou Tong:** Resources. **Shiwei Wei:** Resources. **Naisheng Lu:** Data curation, Methodology, Validation.

## Disclosures

No potential conflict of interest was reported by the authors.
